# Explainable Boosting Machine Predicting Length of Stay After Liver Surgery in Patients with Colorectal Liver Metastases

**DOI:** 10.3390/cancers18132053

**Published:** 2026-06-24

**Authors:** Lucas Alexander Knøfler, Andreas Skov Millarch, Sanne Pagh Møller, Jeanett Klubien, Rasmus Virenfeldt Flak, Claus Wilki Fristrup, Jens Georg Hillingsø, Susanne Dam Nielsen, Martin Sillesen, Henry George Smith, Hans-Christian Pommergaard

**Affiliations:** 1 Department of Digestive Diseases, Transplantation and General Surgery, Copenhagen University Hospital, Rigshospitalet, 2100 Copenhagen, Denmark; lucas.alexander.knoefler@regionh.dk (L.A.K.);; 2 Hepatic Malignancy Surgical Research Unit (HEPSURU), Copenhagen University Hospital, Rigshospitalet, 2100 Copenhagen, Denmark; 3 Section for Enhanced Recovery after Surgery and Disease (ERASD), Copenhagen University Hospital, Rigshospitalet, 2100 Copenhagen, Denmark; 4 National Institute of Public Health, University of Southern Denmark, 1455 Copenhagen, Denmark; 5 Department of Infectious Diseases, Copenhagen University Hospital, Rigshospitalet, 2100 Copenhagen, Denmark; 6 Department of Gastrointestinal Surgery, Aalborg University Hospital, 9000 Aalborg, Denmark; 7 Department of Surgery, Odense University Hospital, 5000 Odense, Denmark; 8 Department for Clinical Medicine, University of Copenhagen, 2200 Copenhagen, Denmark; 9 Abdominalcenter K, Copenhagen University Hospital, Bispebjerg and Frederiksberg, 2000 Frederiksberg, Denmark

**Keywords:** colorectal liver metastases, surgery, length of stay, machine learning, explainable boosting machine

## Abstract

**Background**: Accurate preoperative prediction of length of hospital stay (LOS) after surgery for colorectal liver metastases (CRLMs) could improve patient counselling and resource planning, yet reliable risk tools are lacking. We aimed to develop an interpretable machine learning model predicting LOS following first-time liver-directed surgery for CRLMs. **Methods**: In this multicenter cohort study, we included patients who underwent first-time liver resection, ablation, or a combination for CRLMs at three Danish hepatobiliary centers between 2016 and 2023. Preoperative features from two national registries were used to train Elastic Net, Random Forest, HistGradientBoosting, and Explainable Boosting Machine (EBM) algorithms. Hyperparameters were optimized using five-fold cross-validation. Performance was evaluated on a 20% hold-out test sample using mean absolute error (MAE) with bootstrapped 95% confidence intervals (CIs). **Results**: Among 915 patients, median LOS was 4.0 days (interquartile range (IQR) 3.0–6.0). All four algorithms achieved comparable prediction error (MAE 3.0–3.1 days). The EBM (MAE 3.1 days, 95% CI 2.6–4.3) algorithm was selected for its inherent interpretability. Surgical approach was the strongest predictor, where percutaneous and laparoscopic approaches were associated with reductions of 1.9 and 1.2 days, respectively. Tumor burden, including number of lesions and largest lesion diameter, showed progressive non-linear associations with longer stays. Nonetheless, overall explained variance was low (R^2^ ≤ 0.10), and calibration showed systematic underestimation of stays beyond five days. **Conclusions**: An inherently interpretable machine learning model matched the predictive performance of opaque algorithms for LOS after CRLM surgery, although overall predictive accuracy was modest and longer stays were underestimated. Explainability analysis identified surgical approach and tumor burden as the most influential predictors. External validation in healthcare systems with different discharge practices is warranted.

## Simple Summary

Patients with liver metastases that spread from colon and rectal cancers often undergo surgery as part of their treatment. The length of hospital stay varies considerably and is influenced by factors known before surgery. Predicting this in advance would help surgeons counsel patients, identify those at higher risk, and plan hospital resources, yet no reliable tool currently exists. In this multicenter study of 915 patients treated in Denmark, we developed a transparent prediction model where the reasoning can be audited. Our findings show that the surgical approach and the size and number of tumors were the strongest predictors of a longer stay, while the model itself matched the accuracy of comparable architectures.

## 1. Introduction

Colorectal cancer is the third most common malignancy worldwide and a leading cause of cancer-related death, accounting for approximately 9.3% of global cancer mortality [1]. Nearly a third of patients develop liver metastases within three years of diagnosis [2,3,4]. Modern management of colorectal liver metastases (CRLMs) is multimodal, including systemic therapy, surgical resection, and image-guided thermal ablation [5,6]. In highly selected patients with otherwise unresectable disease, liver transplantation has also emerged as a potentially curative approach [7].

As treatment options continue to evolve, so too must the metrics by which we evaluate them. Length of hospital stay (LOS) is increasingly recognized as a meaningful indicator of perioperative recovery, procedure complications, and resource utilization [8,9]. Despite its relevance, the features associated with LOS after surgery for CRLMs are insufficiently characterized, and reliable preoperative risk tools are lacking. Accurate prediction of LOS has the potential to improve patient counselling, facilitate early identification of high-risk individuals, and support institutional resource planning.

Machine learning (ML) methods offer the capacity to model complex, high-dimensional, and non-linear associations that conventional regression approaches might not capture. However, widespread adoption of ML in clinical practice has been limited by the opacity of many algorithms [10]. In domains such as surgical oncology, where model outputs can directly inform patient care, predictive performance alone is insufficient. Responsible clinical deployment demands interpretability of model reasoning [11,12].

The field of explainable artificial intelligence focuses on model transparency alongside performance. Developers from Microsoft Research advanced this field with the release of the Explainable Boosting Machine (EBM) in 2019 [13]. Built upon the GA2M (Generalized Additive Model with Interactions), the EBM model uses gradient boosting to learn precise, non-linear shape functions for each feature while preserving an additive model structure [14,15,16]. Individual model predictions can be deconstructed into the exact contribution of each preoperative feature, enabling the clinician to understand not only the predicted outcome but also which specific patient and tumor characteristics are driving it.

### 1.1. Primary Objective

To develop and evaluate a machine learning model predicting length of hospital stay following initial liver surgery (resection, ablation, or combined therapy) for colorectal liver metastases.

### 1.2. Secondary Objective

To leverage a model architecture with inherent interpretability to characterize the preoperative clinical features driving predictions at the individual patient level.

## 2. Materials and Methods

### 2.1. Study Design and Data Sources

We conducted a multicenter cohort study of all patients who underwent first-time liver-directed surgery for CRLMs at three of four Danish hepatobiliary centers between 2016 and 2023. Using structural preoperative data, we trained and compared several ML architectures for predicting LOS, evaluated generalizability on a hold-out test sample, and applied explainable artificial intelligence methods to audit model behavior.

Data were collected from two national clinical quality registries. The Danish Liver Cancer Group database (DLGCD) includes patients with primary liver cancer, biliary tract cancer, or liver metastases evaluated at a Danish multidisciplinary team meeting and provides baseline characteristics, procedure details, admission dates, and vital status. The Danish Colorectal Cancer Group database (DCCG) includes records for patients referred for colorectal cancer treatment in Denmark between 2016 and 2022 and provides demographics, comorbidity indices, histopathology, and colorectal cancer surgery details. Individual patient records were linked across registries using the unique civil registration number assigned to all Danish residents.

### 2.2. Study Population

We included all patients aged ≥18 years who underwent their first liver-directed surgery (resection and/or ablation) for CRLM during the study period. One hepatobiliary center was excluded because date of discharge was not recorded, precluding analysis of the target variable.

### 2.3. Predicted Outcome

The primary outcome was LOS, defined as the number of calendar days from the index surgical procedure to hospital discharge.

### 2.4. Clinical Setting and Perioperative Care

In Denmark, treatment of CRLMs is centralized at hepatobiliary units within university-affiliated hospitals. All patients considered for liver surgery are discussed by a multidisciplinary team including liver surgeons, oncologists, hepatologists, and interventional and diagnostic radiologists. If the proposed procedure is deemed technically feasible, the patient is assessed for operability in the outpatient clinic.

Danish healthcare is universally tax-financed, providing all residents with free access to hospital care. Hospitals operate under fixed budgets supplemented by activity-based funding. Perioperative care follows enhanced recovery after surgery (ERAS) principles, including preoperative optimization, early mobilization, and resumption of normal gastrointestinal function [17,18].

### 2.5. Approvals and Ethics

This study was approved by the Danish Health Authority, Capital Region of Denmark (P-2025-18798), and by the DCCG (DCCG-2025-03-31). Informed consent was waived for this study, in accordance with Danish legislation. Reporting adhered to STROBE recommendations and the TRIPOD-AI extension for ML prediction studies [19].

### 2.6. Statistical Methods

#### 2.6.1. Descriptive Analysis

Categorical features are summarized as frequencies and proportions. Normally distributed continuous features are reported as means with standard deviations (SDs) and non-normally distributed features as medians with interquartile ranges (IQRs), assessed by visual inspection of histograms. Descriptive analyses were performed on the complete study cohort prior to data splitting.

#### 2.6.2. Predictors and Preprocessing

All features recorded prior to the index liver surgery were included as candidate predictors. Site-specific features (e.g., hospital center) were excluded to enhance external validity. Liver procedure type (ablation, resection, or combined) and surgical approach (open, laparoscopic, or percutaneous) were extracted from the operative record and therefore correspond to the procedure performed, assuming no deviation from the planned operation. Categorical features were preprocessed using one-hot encoding for the HistGradientBoosting, Random Forest, and Elastic Net algorithms. Missing values in numerical features were imputed using the median, binary features using the mode, and categorical features were assigned a constant label “unknown” to preserve missingness as a potentially informative signal. All imputation was performed exclusively within the training sample to prevent data leakage from the test sample. Standard feature scaling was applied for the Elastic Net regression.

#### 2.6.3. Data Partitioning

The study cohort was randomly partitioned into an 80% training set for model development and a 20% hold-out test set for final performance evaluation. This hold-out evaluation constitutes internal validation, estimating reproducibility within the source population.

#### 2.6.4. Model Strategy and Evaluation

Four learning algorithms were compared: Elastic Net regression as a parametric, fully interpretable baseline, HistGradientBoosting as a regressor, Random Forest as a regressor, and Explainable Boosting Machine as a regressor. We also compared the architectures with simple linear regression restricted to three readily available predictors (age, sex, and procedure type). Hyperparameters were optimized using Optuna’s Tree-structured Parzen Estimator over 100 trials, with performance being assessed by mean absolute error (MAE) under five-fold cross-validation within the training set. Final model performance was evaluated on the hold-out test set. We derived 95% confidence intervals (CIs) for MAE using bias-corrected and accelerated bootstrapping with 10,000 resamples. To characterize prediction error beyond MAE, we also report root mean squared error (RMSE) and the coefficient of determination (R^2^) on the hold-out test set. Model calibration was assessed by plotting actual LOS against predicted LOS for both the training and testing sample.

#### 2.6.5. Explainability Analysis

EBMs fit each feature iteratively in a round-robin fashion using gradient boosting, isolating the contribution of each feature within an additive model structure. Global feature importance was quantified as each feature’s mean absolute contribution to predicted LOS. Shape functions were derived to characterize the direction and magnitude of each feature’s association with LOS while keeping all other features’ effects constant.

#### 2.6.6. Software

All analyses were performed in Python (version 3.13.5). Key libraries included NumPy (version 2.5.0) and Pandas (version 3.0.3) for data handling, Matplotlib (version 3.11.0) and Seaborn (version 0.13.2) for visualization, scikit-learn (version 1.9.0) for preprocessing and model training (Elastic Net, HistGradientBoosting, Random Forest), Optuna (version 4.9.0) for hyperparameter optimization, and InterpretML (version 0.7.8) for the EBM implementation.

## 3. Results

### 3.1. Study Population and Baseline Characteristics

We included 915 patients. The cohort was predominantly male (63.4%) with a median age of 69 years (IQR 60–75). Most patients had significant comorbidity, as 74.9% were classified as ASA II or higher at initial diagnosis. Rectal (33.1%) and sigmoid (29.0%) cancers were the most common primary sites. Advanced colorectal cancer was frequent, with 53.7% classified as pT3 and 59.2% with nodal involvement (pN+) at colorectal resection.

Surgical resection was the most common liver-directed procedure (59.7%), followed by combined resection and ablation (21.3%) and ablation alone (19.0%). An open approach was used in 72.7% of cases. The median interval between colorectal diagnosis and the liver-directed procedure was 4.6 months (IQR 2.5–13.2).

The cohort was randomly partitioned into a training sample (n = 732, 80%) and an independent hold-out test sample (n = 183, 20%). Baseline characteristics are summarized in Table 1.

### 3.2. Length of Stay

**Figure 1:**
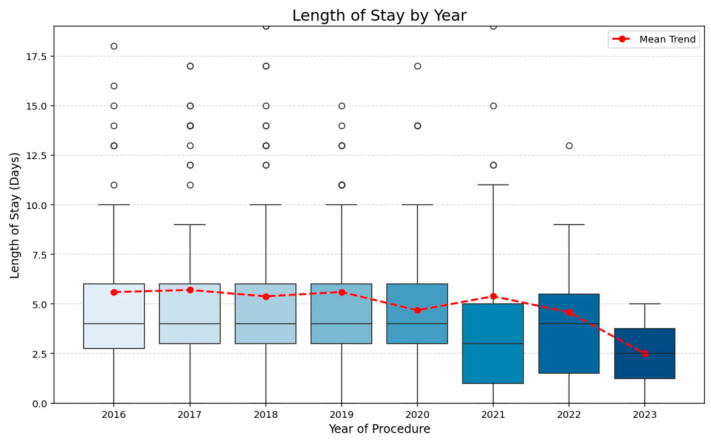
Time trends in length of hospital stay (2016–2023). Box plots with distribution of length of stay (days) for patients undergoing initial liver-directed surgery for colorectal liver metastases, stratified by procedure year. The red dashed line represents the mean length of stay and the grey circles represent outlier observations.

**Figure 2:**
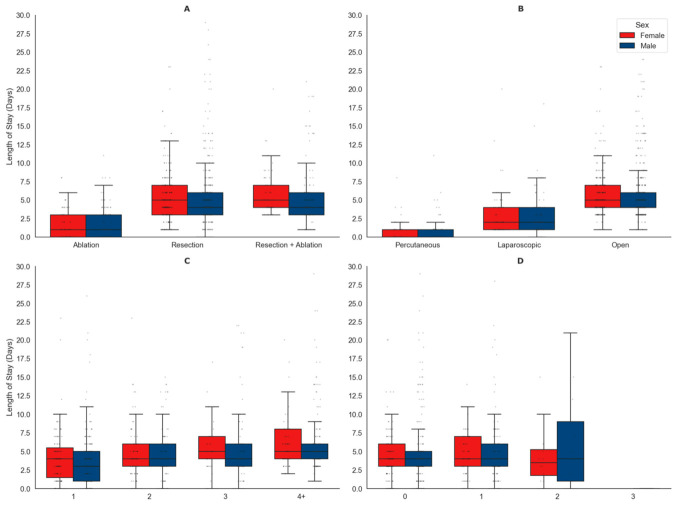
Distribution of length of stay stratified by key clinical features. Box plots showing length of stay (days) according to (**A**) liver procedure type, (**B**) surgical approach, (**C**) number of liver metastases, and (**D**) performance status. The grey dots represent outlier observations. Data are stratified by sex (red = female, blue = male).

The median LOS was 4.0 days (IQR 3.0–6.0), with 97.4% of patients discharged directly to home. Mean LOS declined from 6 days in 2016 to under 4 days by 2023 (Figure 1). Exploratory analysis revealed distinct LOS patterns according to procedural and clinical characteristics. Patients who underwent percutaneous ablation had the shortest stays (median 1 day), whereas open resection or combined therapies had longer, more variable stays (Figure 2A,B). Higher tumor burden (≥4 lesions) was also associated with longer stays (Figure 2C). Distributions of LOS were similar across performance status classes (Figure 2D).

### 3.3. Prediction Error

Table 2 summarizes the prediction error of the four algorithms evaluated on the test sample. Random Forest, HistGradientBoosting, and Elastic Net achieved similar point estimates (MAE 3.0 days), while the EBM was only marginally higher (MAE 3.1 days). Confidence intervals overlapped substantially across all model architectures. Notably, the interval for the EBM (95% CI 2.6–4.3) model largely overlapped those of the remaining algorithms (95% CI ranges from 2.5–4.1 to 2.5–4.2). A simple linear regression restricted to three baseline predictors showed a slightly higher error than ML architectures (MAE 3.3 (95% CI 2.8–4.5), RMSE 6.2, R^2^ −0.02). EBM was selected as the final model based on its inherent interpretability and ability to capture non-linear associations.

### 3.4. Calibration

**Figure 3:**
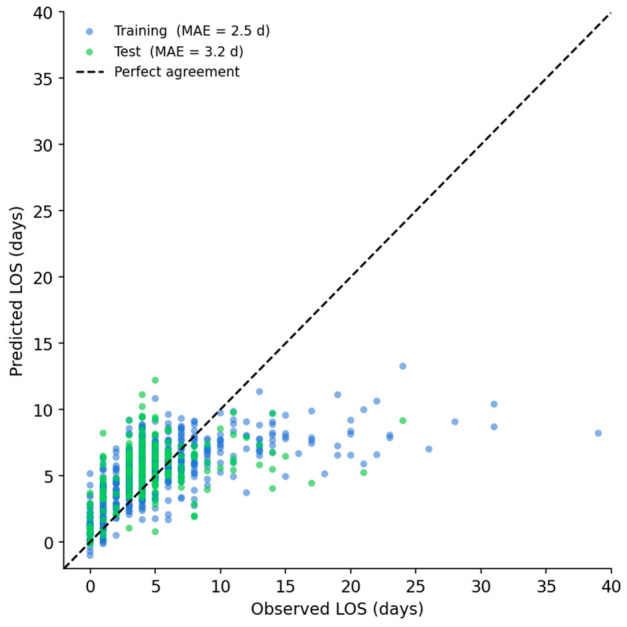
Observed versus predicted length of stay. The horizontal axis shows observed admission time. The vertical axis shows the predicted admission time. Both axes are measured in days. Blue circles represent the 80% training sample, while green circles represent the 20% hold-out test sample. The diagonal dashed line represents perfect agreement between observations and predictions. Abbreviations: MAE = mean absolute error, LOS = length of hospital stay.

Model calibration, assessed by plotting observed against predicted values, showed that for shorter stays (0–5 days), predictions were reasonably well distributed around the line of perfect agreement, with a tendency toward slight overprediction. For admissions exceeding 5 days, the model systematically underestimated LOS (Figure 3).

### 3.5. Global Feature Importance

Global feature importance scores represent each feature’s mean absolute contribution to predicted LOS across the training sample, quantifying how much a given feature shifts the prediction away from the intercept, measured in days.

**Figure 4:**
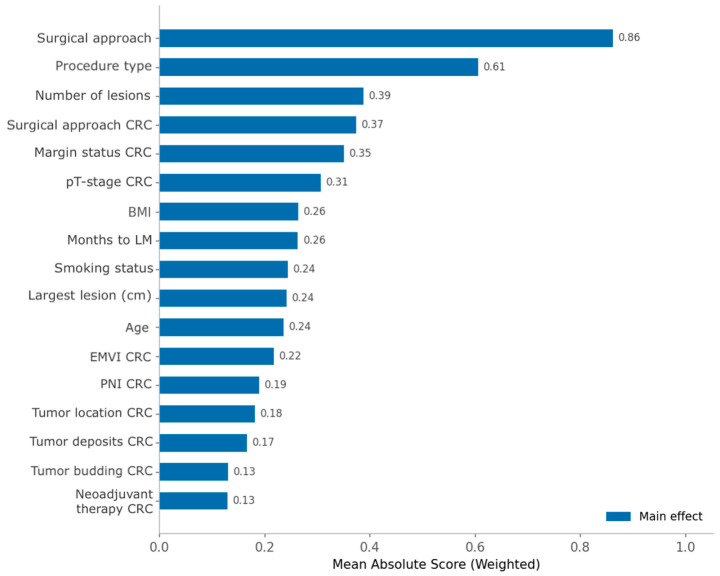
Global feature importance for the Explainable Boosting Machine. The top 15 clinical features ranked by their weighted absolute contribution to the predicted length of stay. The *x*-axis represents the mean absolute score (days), where higher values indicate a stronger influence on predictions. Abbreviations: BMI = body mass index, CRC = colorectal cancer, EMVI = extramural venous invasion, LMs = liver metastases, PNI = perineural invasion, pT = pathological tumor stage.

Characteristics of the liver-directed procedure were most influential. Surgical approach and procedure type shifted predictions by an average of 0.86 and 0.61 days, respectively. Characteristics of the prior colorectal cancer surgery also contributed substantially. Surgical approach, margin status, and pT stage contributed mean absolute values of 0.37, 0.35, and 0.31 days, respectively. Among tumor burden features, the number of liver lesions (0.39 days), time to liver metastases (0.26 days), and size of the largest lesion (0.24 days) were the leading contributors. Patient age, BMI, and smoking status also ranked among the top 11 features (Figure 4). Full importance rankings for all 32 features, including interaction terms, are provided in Supplementary Figure S1.

### 3.6. Shape Functions

Shape functions represent the EBM’s learned contribution of each feature to the predicted LOS, relative to the average contribution across the training sample. Positive values indicate an increase in the predicted LOS relative to the average, and negative values indicate a decrease.

**Figure 5:**
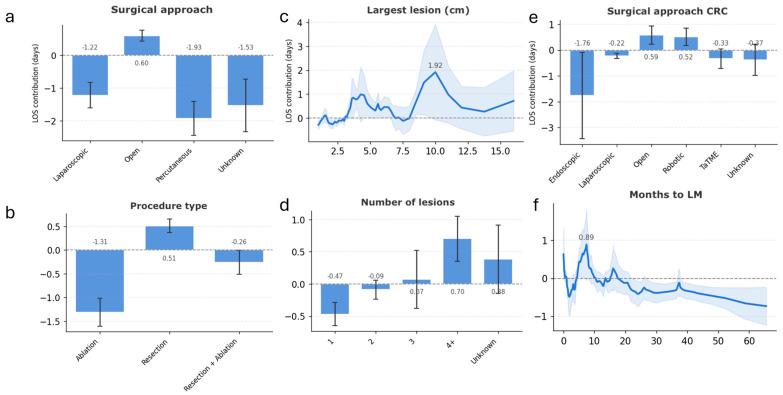
Shape functions for selected top features. Plots show the contribution of features to the predicted length of stay (days), holding all other features constant. (**a**) Surgical approach. (**b**) Procedure type. (**c**) Largest lesion diameter (cm). (**d**) Number of liver lesions. (**e**) Surgical approach colorectal cancer. (**f**) Months to liver metastases. Shaded areas in (**c**,**f**) represents 95% confidence intervals. Abbreviations: CRC = colorectal cancer, LMs = liver metastases, TaTME = transanal total mesorectal excision.

Procedural characteristics showed the strongest differentiation. Open liver surgery contributed 0.6 (±0.2) days relative to the feature average, whereas laparoscopic and percutaneous approaches reduced it by −1.2 (±0.4) and −1.9 (±0.5) days, respectively (Figure 5a). Similarly, an open approach at the time of colorectal cancer resection carried a residual contribution of 0.6 (±0.4) days at the time of subsequent liver surgery (Figure 5e). Liver ablation was associated with a contribution of −1.3 (±0.3) days, whereas resection increased it by 0.5 (±0.1) days (Figure 5b).

The shape function for largest liver lesion diameter suggested a non-linear association. Contributions remained near the feature average for lesions below 3.0 cm, while a progressive rise was seen for larger lesions. The peak was reached at tumor diameters of 9.5 cm with a contribution of 1.9 (CI: −0.1 to 3.9) days, albeit some estimates included 0 (Figure 5c). The number of liver lesions showed a progressive increase, from −0.5 (±0.2) days for a solitary metastasis to 0.7 (±0.4) days for four or more lesions (Figure 5d). For time to liver metastases, an early peak at 7.4 months contributed 0.9 (CI: 0.0 to 1.8) days, followed by a rapid decline and oscillation around the feature average between 10 and 20 months. Beyond 20 months, the shape function crossed below zero and declined steadily, contributing −0.7 (CI: −1.1 to −0.2) days for metastases diagnosed after 54.2 months (Figure 5f).

## 4. Discussion

In this multicenter study of 915 patients undergoing first-time liver-directed surgery for CRLM at Danish hepatobiliary centers, we developed and compared four ML architectures for preoperative prediction of LOS. All algorithms achieved comparable prediction error (MAE 3.0–3.1 days) on a hold-out test sample. The EBM was selected as the final model for inference on the basis of equivalent predictive performance and full interpretability.

Explainability analysis identified the primary drivers that predicted LOS. Surgical approach and procedure type were the dominant contributors. Laparoscopic and percutaneous approaches were associated with substantially shorter stays than open surgery. Tumor burden features, including the number of liver lesions, largest lesion diameter, and time to liver metastases also ranked among the top contributors. Notably, characteristics of the previous colorectal cancer resection, including surgical approach, margin status, and pathological T-stage, carried a residual effect on LOS after liver surgery.

### 4.1. Clinical Interpretation

Surgical approach was the most influential predictor (mean absolute score: 0.86 days), albeit also being the most modifiable predictor. Shape functions showed a lower predicted LOS for percutaneous (−1.9 days) and laparoscopic (−1.2 days) approaches compared with an increase of 0.6 days for open surgery. The direction of these associations is consistent with randomized evidence. The OSLO-COMET trial reported a shorter postoperative hospital stay when patients with CRLM were randomized to laparoscopic rather than open resection [20]. A recent meta-analysis of 35 studies similarly favored minimally invasive surgery over open hepatectomy regarding perioperative complications and LOS [21]. However, these randomized data derive from controlled comparisons and should not be equated with the analysis performed in this study. The choice of surgical approach was determined by the hepatobiliary surgeons and are strongly influenced by tumor complexity, anatomical location, and patient selection. The modeled contribution of surgical approach may reflect the case mix as much as it reflects the procedure itself.

Hepatic resection has historically been considered the gold standard for curative treatment of CRLMs, while thermal ablation was reserved for patients unfit for resection. Recent evidence challenges this notion. The MAVERRIC trial reported less treatment-associated morbidity and comparable overall survival with ablation compared with resection [22]. An umbrella review of 11 meta-analyses demonstrated a lower risk of postoperative complications following ablation, albeit with higher intrahepatic recurrence and lower disease-free survival [23]. The EBM’s shape function for procedure type aligns well with these findings. Ablation was associated with a reduction of −1.3 days in predicted LOS, whereas resection increased it by 0.5 days. Given the broad foundation of preoperative features used for training, the model likely captures real-world treatment allocation patterns in which ablation may also be used for frail and unresectable patients but still results in shorter stays.

The surgical approach used for colorectal cancer surgery also influenced predicted LOS at the time of liver surgery (mean absolute score: 0.37 days). The model identified a carried residual risk where previous open colorectal surgery contributed 0.6 days to LOS. Previous research demonstrates that open surgery increases the incidence of adhesions to the ventral abdominal wall [24]. Adhesions often necessitate tedious dissection and prolong both operative and recovery time. They may also result in conversion from minimally invasive to open surgery [25,26].

Metastatic burden was also a top-tier driver of LOS. The model attributed a reduction of −0.5 days for solitary liver lesions and a stepwise increase to 0.7 days for patients with four or more lesions, and despite uncertain intervals, it is consistent with longer operative times and greater perioperative blood loss [27]. The shape function for largest lesion diameter suggested a non-linear association, with contributions rising progressively above 3 cm, although the magnitude at the largest diameters was imprecisely estimated (intervals including zero). This tipping point may represent a transition from parenchymal-sparing procedures such as ablation and wedge resections to larger anatomical resection with increased risk of complications and thus longer LOS [28].

Predictive performance and interpretability in ML have traditionally been considered a trade-off [12]. Our results suggest otherwise. The EBM and Elastic Net models differed by only 0.1 days in MAE, indicating a negligible performance delta. The EBM offers inherently interpretable shape functions, in which each feature is trained iteratively using gradient-boosted trees [13]. In contrast, Random Forest and HistGradientBoosting require post hoc approximations to distribute credit to individual features [29], and Elastic Net cannot model non-linear associations. In clinical settings where model outputs may inform patient care, this interpretability–performance balance is a meaningful advantage [11].

Calibration analysis showed that predictions were most accurate for stays of 0–5 days, with systematic underestimation of longer admissions. This underestimation may have several explanations. The model was tuned to minimize mean absolute error. Because the LOS distribution is right-skewed, this criterion targets the median stay and pulls predictions below the longest stays. Furthermore, it is reasonable to assume that postoperative LOS is not exclusively determined by preoperative factors. Stochastic events, including intraoperative and postoperative adverse events, and discharge logistics inevitably contribute to the eventual outcome and place a ceiling on what can be forecast from preoperative information. In keeping with this, the proportion of variance explained was low for every algorithm (R^2^ ≤ 0.10 on the hold-out test sample), substantiating that preoperative information alone accounts for only a small fraction of the variation in LOS, at least for the prolonged stays that are of greatest interest for risk stratification and resource management. The observed MAE of 3.0–3.1 days represents this inherent uncertainty. To the best of our knowledge, no directly comparable model for LOS after liver-directed surgery for CRLMs has been published. A relevant benchmark is provided by a 2025 study of patients undergoing hepatectomy, which reported MAEs of 1.15 days for minor and 1.38 days for major resection using a comparable model architecture. However, those models were trained on intraoperative and postoperative features in addition to preoperative features. The accuracy gap between that work and the proposed EBM is likely attributable to the capture of intraoperative and postoperative events [30].

### 4.2. Strengths and Limitations

A major strength of this study is the use of nationwide, multicenter registry data from the Danish Liver Cancer Group and the Danish Colorectal Cancer Group. This provided a wide range of preoperative features spanning the primary colorectal cancer, metastatic disease, and liver surgery. The application of the EBM model addresses a critical barrier to integration of ML in clinical practice. By demonstrating that a fully interpretable architecture achieves performance comparable with conventional boosting and bagging algorithms, we can provide a model whose reasoning can be explicitly audited by clinicians.

Several limitations merit discussion. First, we restricted the model to preoperative features available right before the time of surgery. The model therefore does not account for intraoperative events such as blood loss, operative time, or conversions to open surgery. This was a deliberate design choice, as we aimed to develop a model relevant at the point of preoperative counselling, likely at the expense of predictive precision. Certainly, it must be also acknowledged that the underestimation of patients with longer LOS constitute the subgroup of greatest clinical interest for preoperative risk prediction. Second, readmission data were unavailable, precluding assessment of whether shorter predicted stays correspond to safe long-term recovery or premature discharge. Third, the study population was drawn exclusively from a universal, tax-funded Danish healthcare system with no financial incentives tied to discharge timing. Model evaluation was assessed by internal validation using a random data split. Because both partitions were drawn from the same health system and same calendar period, results may not generalize to other healthcare systems.

Several features had substantial missing data, most notably tumor budding (41.7%), performance status (32.2%), and lymphatic invasion (32.2%). Numerical features were imputed by the training set median and binary features by the mode, while missing categorical values were retained as a distinct category, allowing the models to exploit potentially informative missingness. The dominant predictors were almost completely recorded (<2.3% missing), whereas high rates of missing values were concentrated among lower-ranked features.

## 5. Conclusions

Using preoperative data to predict LOS after first-time liver surgery for CRLMs, we demonstrate that an inherently interpretable ML architecture achieved the same prediction error as other more opaque algorithms. Surgical approach and procedure type were the most influential predictors, with minimally invasive and ablation techniques associated with shorter predicted stays. Overall predictive accuracy was nonetheless modest, with low explained variance and systematic underestimation of longer admissions. External validation of our findings is warranted to confirm generalizability across healthcare systems.

## Figures and Tables

**Table 1:** Baseline characteristics of the study population. The cohort is randomly partitioned between a training sample (n = 732, 80%) for model training and an independent hold-out test sample (n = 183, 20%) for evaluation. Abbreviations: ADC = adenocarcinoma, ASA = American Society of Anesthesiologists, BMI = body mass index, dMMR = deficient mismatch repair, IQR = interquartile range, NOS = not otherwise specified, SD = standard deviation, TaTME = transanal total mesorectal excision. * Feature excluded from model training.

Characteristic	All Patients (n = 915)	Training (n = 732)	Testing (n = 183)
Patient Demographics and Baseline Characteristics			
Male—no. (%)	580 (63.4)	459 (62.7)	121 (66.1)
Median age (IQR)	69 (60–75)	69 (60–75)	67 (59–75)
BMI, kg/m^2^			
Mean (SD)	22.7 (4.6)	22.6 (4.5)	23.0 (5.0)
Missing—no. (%)	18 (2.0)	15 (2.1)	3 (1.6)
ASA classification—no. (%)			
I	213 (23.3)	172 (23.5)	41 (22.4)
II	546 (59.7)	432 (59.9)	114 (62.3)
III	139 (15.2)	113 (15.4)	26 (14.2)
IV	1 (0.1)	1 (0.1)	0 (0)
Missing	16 (1.7)	14 (1.9)	2 (1.1)
Performance status—no. (%)			
0	374 (40.9)	294 (40.2)	80 (43.7)
1	214 (23.4)	172 (23.5)	42 (23.0)
2	29 (3.2)	27 (3.7)	2 (1.1)
3	3 (0.3)	3 (0.4)	0 (0)
Missing	295 (32.2)	236 (32.2)	59 (32.2)
Alcohol intake—no. (%)			
1–14 units/week	557 (60.9)	452 (61.7)	105 (57.4)
15–21 units/week	48 (5.2)	38 (5.2)	10 (5.5)
>21 units/week	47 (5.1)	37 (5.1)	10 (5.5)
Missing	263 (28.7)	205 (28.0)	58 (31.7)
Smoking status—no. (%)			
Never smoked	356 (38.9)	281 (38.4)	75 (41.0)
Previous smoker	289 (31.6)	235 (32.1)	54 (29.5)
Smoker	165 (18.0)	127 (17.3)	38 (20.8)
Missing	105 (11.5)	89 (12.2)	16 (8.7)
Characteristics of Liver Metastases			
Median time to liver metastases (IQR)	4.6 (2.5–13.2)	4.6 (2.4–13.1)	5.0 (2.6–13.4)
Median diameter of largest metastasis—cm. (IQR)	2.4 (1.6–3.2)	2.3 (1.6–3.1)	2.5 (1.7–3.5)
Missing—no. (%)	128 (14.0)	101 (13.8)	27 (14.8)
Number of liver metastases—no. (%)			
1	336 (36.7)	274 (37.4)	62 (33.9)
2	197 (21.5)	158 (21.6)	39 (21.3)
3	128 (14.0)	100 (13.7)	28 (15.3)
4+	233 (25.5)	182 (24.9)	51 (27.9)
Missing	21 (2.3)	18 (2.5)	3 (1.6)
Procedure type—no. (%)			
Ablation	174 (19.0)	138 (18.9)	36 (19.7)
Resection	546 (59.7)	434 (59.3)	112 (61.2)
Ablation + resection	195 (21.3)	160 (21.9)	35 (19.1)
Surgical approach—no. (%)			
Percutaneous	110 (12.0)	91 (12.4)	19 (10.4)
Laparoscopic	139 (15.2)	114 (15.6)	25 (13.7)
Open	665 (72.7)	526 (71.9)	139 (76.0)
Missing	1 (0.1)	1 (0.1)	0 (0)
Median length of stay (IQR)	4.0 (3.0–6.0)	4.0 (3.0–6.0)	4.0 (3.0–6.0)
Discharge destination—no. (%) *			
Home	891 (97.4)	-	-
Other hospital	15 (1.6)	-	-
Deceased	2 (0.2)	-	-
Missing	7 (0.8)	-	-
Characteristics of Colorectal Cancer (Primary Tumor)			
Tumor location—no. (%)			
Caecum	95 (10.4)	80 (10.9)	15 (8.2)
Ascending colon	66 (7.2)	51 (7.0)	15 (8.2)
Hepatic flexure	28 (3.1)	22 (3.0)	6 (3.3)
Transverse colon	42 (4.6)	28 (3.8)	14 (7.7)
Splenic flexure	22 (2.4)	16 (2.2)	6 (3.3)
Descending colon	35 (3.8)	30 (4.1)	5 (2.7)
Sigmoid colon	265 (29.0)	215 (29.4)	50 (27.3)
Rectum	303 (33.1)	245 (33.5)	58 (31.7)
Colon NOS	59 (6.4)	45 (6.1)	14 (7.7)
Screening diagnosis—no. (%)	183 (20.0)	146 (19.9)	37 (20.2)
Histological subtype—no. (%)			
ADC	670 (73.2)	541 (73.9)	129 (70.5)
High-grade ADC	39 (4.3)	25 (3.4)	14 (7.7)
Mucinous ADC	49 (5.4)	40 (5.5)	9 (4.9)
Signet Ring	1 (0.1)	1 (0.1)	0 (0)
Missing	156 (17.0)	125 (17.1)	29 (15.8)
Extramural venous invasion—no. (%)	393 (43.0)	309 (42.2)	84 (45.9)
Missing	143 (15.6)	113 (15.4)	30 (16.4)
Perineural invasion—no. (%)	209 (22.8)	175 (23.9)	34 (18.6)
Missing	160 (17.5)	130 (17.8)	30 (16.4)
Lymphatic invasion—no. (%)	156 (17.0)	129 (17.6)	27 (14.8)
Missing	295 (32.2)	243 (33.2)	52 (28.4)
dMMR—no. (%)	29 (3.2)	20 (2.7)	9 (4.9)
Missing	103 (11.3)	87 (11.9)	16 (8.7)
Tumor budding—no. (%)	190 (20.8)	157 (21.4)	33 (18.0)
Missing	382 (41.7)	305 (41.7)	77 (42.1)
Tumor deposits—no. (%)	230 (25.1)	179 (24.5)	51 (27.9)
Missing	152 (16.6)	121 (16.5)	31 (16.9)
Tumor perforation—no. (%)	27 (3.0)	21 (2.9)	6 (3.3)
Missing	189 (20.7)	154 (21.0)	35 (19.1)
Characteristics of Colorectal Cancer (Primary Tumor)			
Surgical approach—no. (%)			
Endoscopic	13 (1.4)	10 (1.4)	3 (1.6)
Laparoscopic	476 (52.0)	382 (52.2)	94 (51.4)
Robotic	129 (14.1)	110 (15.0)	19 (10.4)
Open	177 (19.3)	136 (18.6)	41 (22.4)
TaTME	21 (2.3)	16 (2.2)	5 (2.7)
Missing	99 (10.8)	78 (10.7)	21 (11.5)
Acute surgery—no. (%)	62 (6.8)	52 (7.1)	10 (5.5)
Missing	99 (10.8)	78 (10.7)	21 (11.5)
Neoadjuvant therapy—no. (%)			
Chemotherapy	216 (23.6)	164 (22.4)	52 (28.4)
Chemoradiotherapy	75 (8.2)	69 (9.4)	6 (3.3)
Radiotherapy	11 (1.2)	10 (1.4)	1 (0.5)
None	514 (56.2)	411 (56.1)	103 (56.3)
Missing	99 (10.8)	78 (10.7)	21 (11.5)
Peritoneal involvement—no. (%)	151 (16.5)	125 (17.1)	26 (14.2)
Missing	141 (15.4)	111 (15.2)	30 (16.4)
Involvement of other organs—no. (%)	41 (4.5)	33 (4.5)	8 (4.4)
Missing	143 (15.6)	114 (15.6)	29 (15.8)
Pathological T-stage—no. (%)			
pT0	13 (1.4)	10 (1.4)	3 (1.6)
pT1	14 (1.5)	13 (1.8)	1 (0.5)
pT2	80 (8.7)	62 (8.5)	18 (9.8)
pT3	491 (53.7)	391 (53.4)	100 (54.6)
pT4	178 (19.5)	146 (19.9)	32 (17.5)
Missing	139 (15.2)	110 (15.0)	29 (15.8)
Pathological N-stage—no. (%)			
pN0	234 (25.6)	189 (25.8)	45 (24.6)
pN1	315 (34.4)	253 (34.6)	62 (33.9)
pN2	227 (24.8)	180 (24.6)	47 (25.7)
Missing	139 (15.2)	110 (15.0)	29 (15.8)
Anastomotic leak—no. (%)	31 (3.4)	24 (3.3)	7 (3.8)
Missing	112 (12.2)	88 (12.0)	24 (13.1)
Residual tumor status—no. (%)			
R0	657 (71.8)	518 (70.8)	139 (76.0)
R1	51 (5.6)	43 (5.9)	8 (4.4)
R1LNM	60 (6.6)	54 (7.4)	6 (3.3)
Missing	147 (16.1)	117 (16.0)	30 (16.4)

**Table 2:** Prediction error on hold-out test sample. Metrics calculated on the 20% hold-out sample (n = 183) using MAE, RMSE, and R^2^. The 95% confidence intervals estimated using bias-corrected and accelerated bootstrapping with 10,000 resamples. Abbreviations: CI = confidence interval, EBM = Explainable Boosting Machine, MAE = mean absolute error, RMSE = root mean squared error, R^2^ = coefficient of determination.

Algorithm	MAE	95% CI	RMSE	R^2^
Random Forest	3.0	2.5–4.2	5.9	0.07
HistGradientBoosting	3.0	2.5–4.2	6.0	0.06
EBM	3.1	2.6–4.3	6.0	0.03
Elastic Net	3.0	2.5–4.1	5.8	0.1

## Data Availability

The data presented in this study are only available upon approved request from the Danish Liver Cancer Group (DLGCD) and the Danish Colorectal Cancer Group (DCCG).

## Supplementary Materials

The following supporting information can be downloaded at: https://www.mdpi.com/article/10.3390/cancers18132053/s1, Figure S1: Global feature importance for the Explainable Boosting Machine.
