# Supplementary material for: Explainable Boosting Machine Predicting Length of Stay After Liver Surgery in Patients with Colorectal Liver Metastases
Source: Cancers (Basel). 2026 Jun 24;18(13):2053. doi: 10.3390/cancers18132053 (PMC13359731; doi:10.3390/cancers18132053)
Supplement: Supplementary file 1 [file cancers-18-02053-s001.zip › cancers-4329474-supplementary.pdf]

cancers MDPI

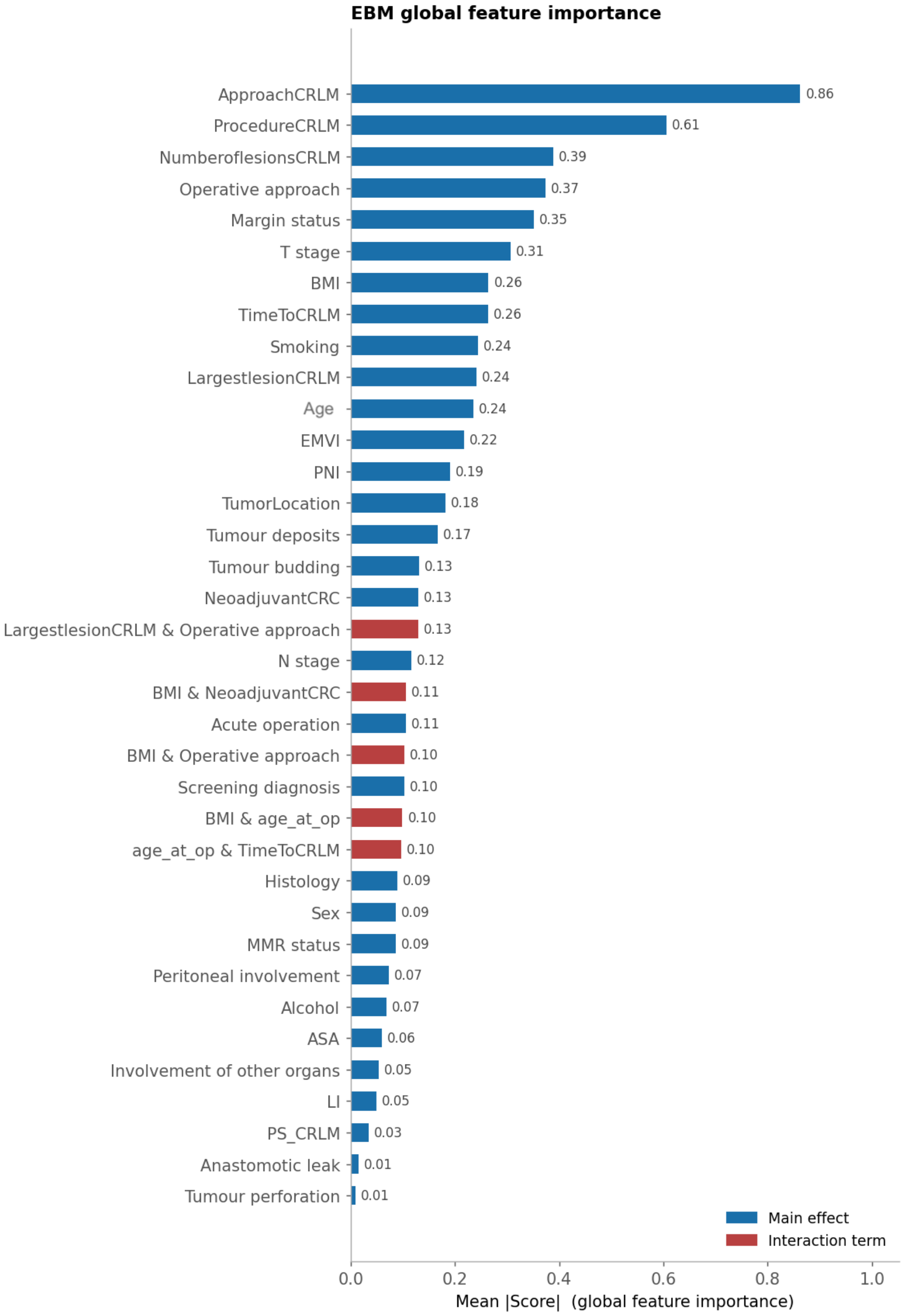

Figure S1. Global feature importance for the Explainable Boosting Machine. Full list of clinical features ranked by their weighted absolute contribution to the predicted length of stay. The x-axis represents the mean absolute score (days), where higher values indicate a stronger influence on predictions. **Abbreviations:** ASA = American Society of Anesthesiologists Physical Status Classification, BMI = body mass index, CRC = colorectal cancer, EMVI = extramural venous invasion, LI = lymphovascular invasion, LM = liver metastases, MMR = mismatch repair, N stage = pathological nodal stage, PNI = perineural invasion, PS_CRLM = performance status at liver surgery, pT = pathological tumor stage.
